# The Unusual Circulation of the Newt Heart after Ventricular Injury and Its Implications for Regeneration

**DOI:** 10.1155/2011/812373

**Published:** 2011-09-15

**Authors:** Yukihisa Miyachi

**Affiliations:** Laboratory for Occupational Safety and Health, National Cerebral and Cardiovascular Center Research Institute, Fujishirodai 5-7-1, Suita, Osaka 565-8565, Japan

## Abstract

Why do newts survive after needle puncture of the heart despite significant hemorrhage into the thoracic cavity? The answer involves the unique anatomical changes in the circulation that occur after ventricular injury. If the ventricle ruptures, newts quickly develop valve hyperplasia at the location of both the ventricular inflow and outflow tracts so as to redirect blood flow away from the injured ventricle. In addition, there is collateral flow between the left anterior caval vein and the conus arteriosus (a part of the aorta) after ventricular injury that supplements the systemic circulation and helps maintain vital organ perfusion. During this time period, the damaged ventricle can regenerate.

## 1. Introduction

Many studies of newts support the notion that regeneration is largely achieved via a dedifferentiation process [1, 2]. For example, the transplantation of fluorescent-labeled cardiomyocytes into regenerating limb blastema results in the downregulation of cardiac marker genes and the upregulation of undifferentiated genes, which indicate that these cells can dedifferentiate [3]. I reported that lens regeneration in the newt requires the destruction and engulfment of damaged tissue by immune cells. I hypothesize that VEGFR expression by antigen-presenting cells is essential for angiogenesis and the subsequent regeneration [4].

In the newt, generally, the regeneration begins in a different site from the location where the organ was removed surgically. For example, lens regeneration begins in the iris or cornea. I performed an experiment in which the newt was observed to generate cardiomyocytes after cardiac injury; this process was similar to the induction of experimental lens regeneration. During this experiment, I noticed that the newts did not die even though they had severe ventricular injury and that a significant amount of blood was stored in their thoracic cavities. To understand newt survival after ventricular injury and failure, I examined the possible mechanisms by which these animals maintain circulatory function while cardiac regeneration occurs.

## 2. Methods

Adult newts, *Cynops pyrrhogaster*, were reared in polycarbonate cages in an environmentally controlled room (water temperature: 22 ± 1°C), with a standard 12-hour light/dark cycle. The hearts from 600 newts were pricked twice with a needle (18 G syringe) through the skin to induce injury. Chest portions were fixed for 24 hours in a 10% formalin solution that was buffered with 0.15 M sodium-phosphate at a pH of 7.3. They were then washed in several changes of the same buffer and embedded in paraffin. Serial sections of 2 *μ*m were prepared and stained with Mayer's hematoxylin and eosin, and the degree of heart damage and subsequent remodeling were evaluated. Masson's trichrome stain (acid fuchsin-orange G and aniline blue stain) was used for the characterization of collagen fibrogenesis, and alcian blue or toluidine blue was used to stain chondrocytes. The immunohistochemistry was used to detect CD31, similar to that described by Cursiefen et al. [5]. Briefly, the chest portion of each newt was frozen on dry ice in Tissuetek and sectioned into 8-*μ*m thick slices. The sections were collected onto microscope slides, dried at room temperature, fixed in acetone, rinsed in PBS, blocked in 2% BSA, and stained with horseradish-peroxidase- (HRP-) conjugated CD31 antibody overnight. All staining procedures were performed at room temperature. Staining with secondary antibody alone, or with an isotype control instead of with CD31 primary antibody, was negative. Cell death was indicated by the expression of LC3 protein, which is a protein associated with autophagocytosis. All experiments were approved by the animal ethics committee of the National Cerebral and Cardiovascular Center.

## 3. Results

### 3.1. Heart Injury

**Figure 1:**
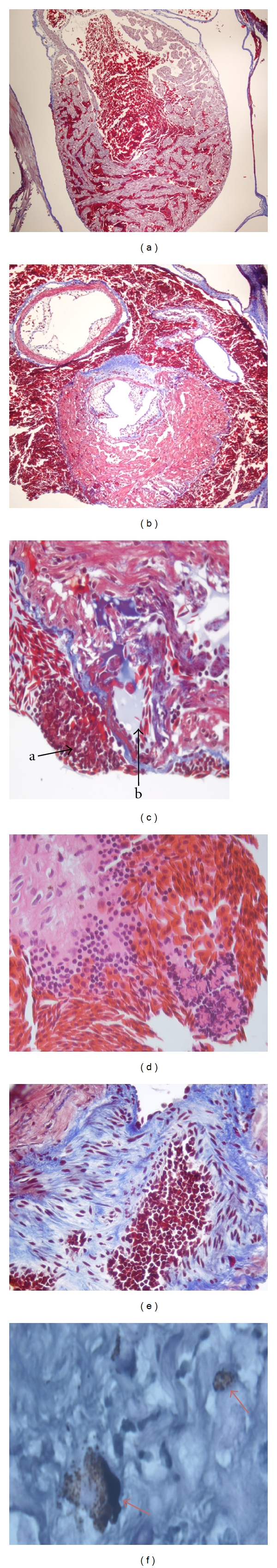
Bleeding from the heart after needle puncture. (a) An intact newt ventricle and (b) a ventricle that is surrounded by blood. (c) Location of the needle-stick in the heart (arrow “b”). Arrow “a” indicates a coagulated clot. Sections (a–c) were processed with Masson's trichrome stain. (d) A ventricle that is surrounded by phagocytes (HE stain). (e) Collagen fibrogenesis of cardiomyocytes after needle puncture (trichrome stain). The collagenous fibers are stained blue. (f) Expression of LC3, a protein associated with autophagic cell death. LC3 protein that is conjugated with HRP-black is observed (arrows). Magnification: ((a), (b), and (d)) ×40; ((c), (e), and (f)) ×100.

It is well known that amphibious hearts consist of two atria and one ventricle, which differ from the two atriums and two ventricles found in mammalian hearts. When I injured the newt ventricle by puncture with a thick needle, as shown in Figure 1, many erythrocytes escaped from the wound and surrounded the injured heart (Figure 1(b)). In contrast, erythrocytes were only observed in the ventricular lumen of control specimens (Figure 1(a)). After needle puncture, blood coagulation was observed around the ventricular wound (Figure 1(c)). Neutrophilic cells important for the removal of damaged tissue were seen in the thoracic cavity (Figure 1(d)). 

**Figure 2:**
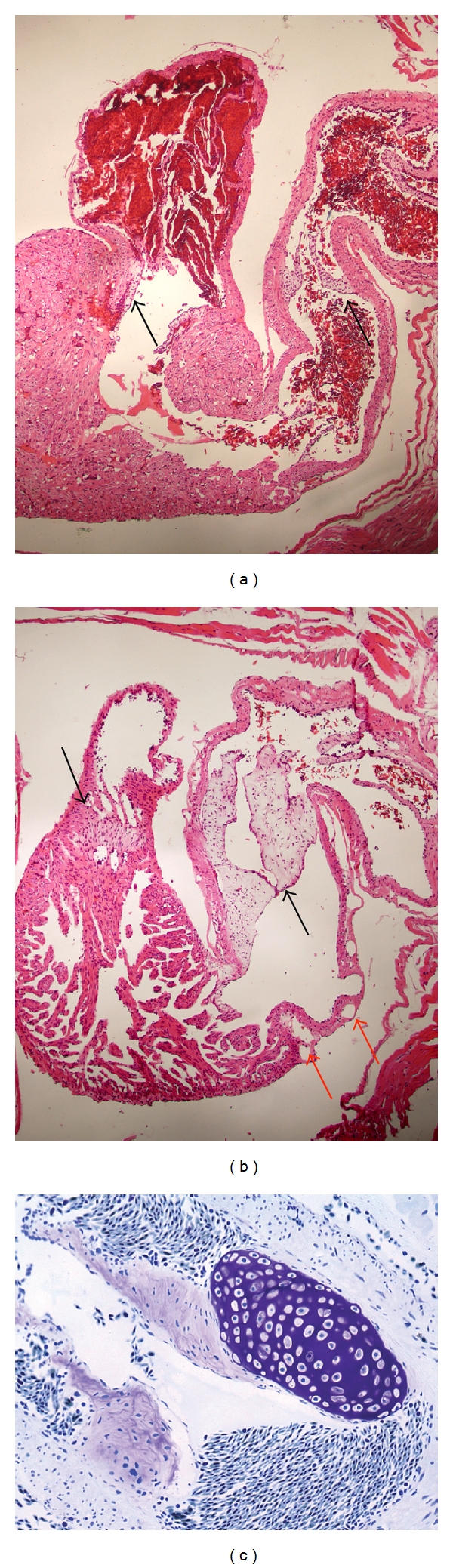
Valve abnormalities during regeneration. (a) The heart of an intact animal (HE stain). Blood is shown in the left atrium. The blood is just about to enter the ventricle. The valve is completely open (arrows). (b) Hypertrophy of the valve after needle puncture. The black arrows indicate the valves. Note that the blood is not shown in the ventricle after needle puncture (HE stain). The red arrows indicate the coronary vessels on the conus arteriosus. (c) Chondrogenesis started in the valve in the location of the conus arteriosus. The violet-colored tissue stained by toluidine blue is cartilage. Magnification: ((a) and (b)) ×40; (c) ×100.

At this point in time, a remarkable change occurred in the valves (Figure 2). The animals with ventricular puncture exhibited valve hypertrophy in the conus arteriosus (a part of the main outflow tract).  Figure 2(a) shows blood outflow to the conus arteriosus with the contraction of the ventricle in uninjured newts.  Figure 2(b) indicates that blood outflow was blocked by the hypertrophic valve after needle puncture, and cardiomyocytes were also shed from the heart. When the valves were stained with toluidine blue, I found that the cells in the valve endoskeleton exhibited metachromasia (Figure 2(c)). This finding indicates that chondrocytes began to form in the valve. Hyperplasia was also observed in the valve between the left atrium and the ventricle (arrow in Figure 2(b)).

In the next few days, the beginning of scar formation was confirmed at the wound site using acid trichrome staining. In addition, expression of LC3 protein occurred around these damaged tissues (Figure 1(f)), suggesting that cell death occurred by autophagocytosis.

### 3.2. Newt Blood Circulation

**Figure 3:**
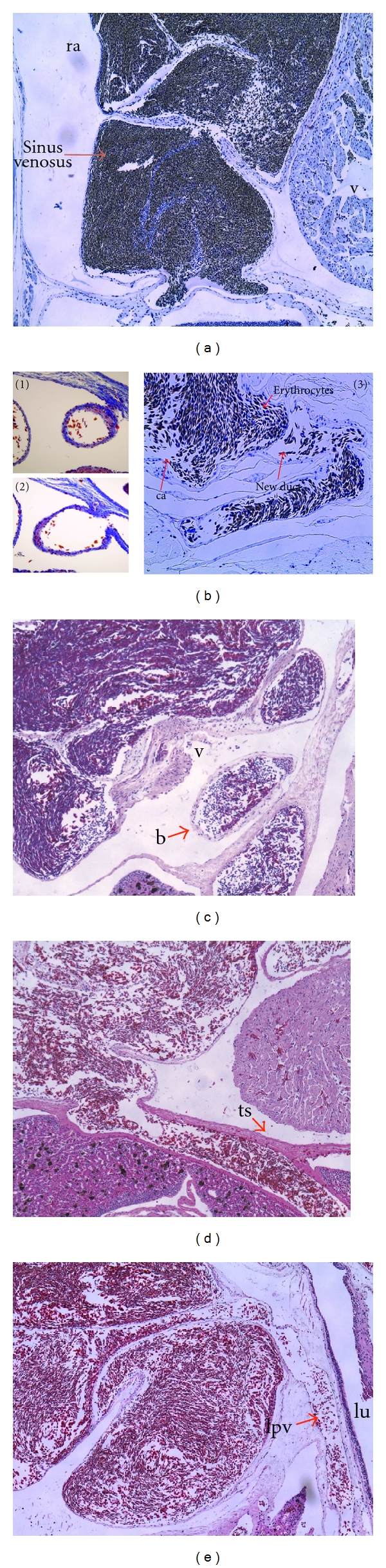
Changes in the circulatory system after needle puncture. (a) The sinus venosus around the right atrium (HE stain). The acronym “ra” stands for right atrium and “v” for ventricle. (b) The new duct between the left anterior cava vein and the conus arteriosus. (1) 2 hours, (2) 6 hours, and (3) 12 hours after needle puncture (trichrome stain). (c) A bulge is shown in a section from the side. The acronym “b” stands for a bulge and “v” for ventricle. (d) A duct under the transverse septum (HE stain). The acronym “ts” stands for transverse septum. (e) The left pulmonary artery. This section is viewed from the side (HE stain). The acronym “lu” stands for left lung and “lpv” for left pulmonary vein. Magnification: ((a), (b)-(3), (c), (d), and (e)) ×40; ((b)-(1) and (b)-(2)) ×100.

I examined the circulation of the intact newt heart, since there is not much information on this topic in the published literature. The anatomical results showed that blood flowed from the peripheral veins including the anterior and posterior caval veins (Figure 3(d)) into the sinus venosus. In addition, two pulmonary veins (Figure 3(e)) deliver blood into the sinus venosus (Figure 3(a)). Blood flows from the sinus venosus into the right atrium. After that, blood that went to the left atrium moves to the ventricle and is expelled out towards the conus arteriosus. Efficient contraction of the heart was observed at the location of the sinus venosus, the two atria, the ventricle, and the conus arteriosus. These observations suggest that the cardiac impulse propagates from the sinoatrial node to the atrioventricular node. When I placed a ligature between the sinus venosus and the right atrium, the newt died. However, the newts that had needle-induced rupture of the sinus venosus survived. This difference indicates that the sinoatrial node is located in the sinus venosus. A coronary circulation was observed only in the conus arteriosus (red arrow in Figure 2(b)), indicating that the newt does not have a coronary artery on the ventricle.

When I injured the ventricle, a small-diameter duct developed between the left anterior caval vein and the conus arteriosus within 12 h of needle puncture (Figure 3(b)). This duct enabled blood that was originally flowing into the ventricle to flow instead directly into the ascending aorta. This collateral flow reduced the amount of hemorrhage from the ventricular puncture site and gave time for the injured heart to regenerate. 

Other changes could be observed in the duct derived from the left superior caval vein. Part of the duct was remarkably expanded and the blood temporarily gathered in this expanded duct over the transverse septum. This blood was rhythmically forced towards the vessel that runs under the transverse septum. This side of the bulge is shown in Figure 3(c), and histology showed that the bulge was an independent structure. Blood may be stored temporarily in the bulge because blood flow toward the ventricle is restricted by valve hyperplasia. The blood from the bulge was observed to mix with blood from the vein draining the liver immediately after penetrating the transverse septum.

### 3.3. Repair and Regeneration

**Figure 4:**
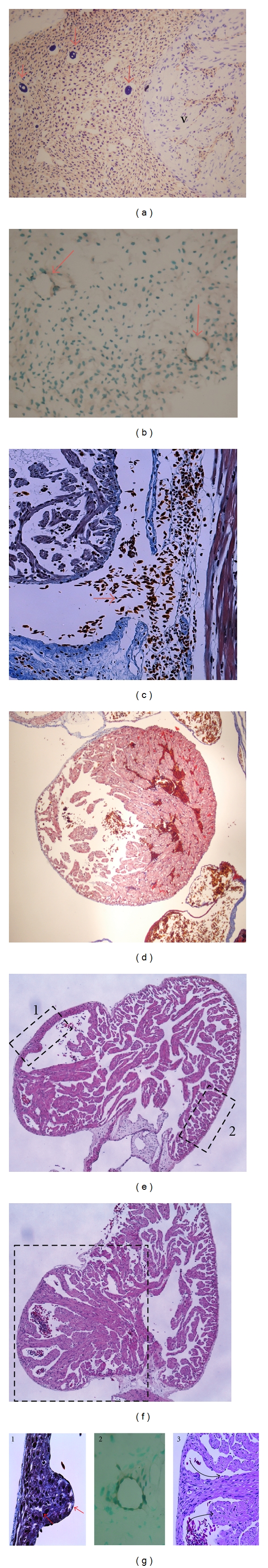
Regeneration after needle puncture. (a) Chondrogenesis is shown based on toluidine blue staining. (b) New vessels are shown by CD31 immunohistological staining. (c) Formation of a fistula (toluidine blue). (d) There is loss of cardiomyocytes after needle puncture (trichrome stain). (e) Regenerating cardiomyocytes 7 days after needle puncture. Note that the epicardial layer where regeneration starts is significantly thickened (box 1, HE stain) compared with areas where there is no regeneration (box 2, HE stain). (f) Regenerating cardiomyocytes 14 days after needle puncture. The rectangular box shows regenerating cardiomyocytes. (g) Panel 1 is a magnification of the rectangular space-1 in (e). The red arrows indicate the blood vessels (trichrome stain). Immunohistological analysis showed that CD31 is expressed in the tip of the epicardial bud (panel 2). Cardiomyocyte regeneration required more time in the endocardial layer (panel 3, HE stain). Magnification: ((d), (e) and (f)) × 40; ((a), (b), (c) and (all panels of g)) × 100.

I found that cells from the blood clot in the thoracic cavity showed metachromasia when the cells were stained with toluidine blue (Figure 4(a)). To address the question of whether vascularization may have occurred with the onset of regeneration, immunohistochemical staining for CD31 was performed. Several round-shaped structures surrounding the site of cardiac injury were clearly visible (Figure 4(b)). This indicates that a network of neovascularization formed outside of the heart prior to regeneration and the new vessels might play an essential role in the regenerative process. 

The clotted blood observed in the thoracic cavity disappeared within 12 hours after the puncture was made. I considered the possible ways in which the clotted blood could have been removed. I observed a new path in the pleura of the thoracic cavity that allowed the extrusion of the clot remains (Figure 4(c)). The posterior caval vein that runs outside of the thoracic cavity was extended due to the entry of the clot remains.

At the beginning of regeneration, the epicardial layer around the site of injury gradually thickened (box 1 in Figure 4(e)) compared with the layers away from the injury site (box 2 in Figure 4(e)). After that, part of the epicardial layer protruded like a bud (Figure 4(g)-1). The new vessels were found at site of epicardial protrusion (Figure 4(g)-2). The regeneration of cardiomyocytes extended toward the endocardium and finally there was regeneration of the entire myocardial wall (Figures 4(f) and 4(g)-3). There was a clear difference between the regenerated hearts compared with hearts observed immediately after injury (Figure 4(d)). 

## 4. Conclusion

How do newts survive ventricular puncture that causes hemorrhage into the thoracic cavity? The answer lies in the unique adaptive changes in the circulatory system that develop rapidly after ventricular injury. When I punctured the ventricle with a needle, there was immediate collateral blood flow between the left anterior cava vein and the conus arteriosus. This duct allowed blood to be diverted away from the injured ventricle and flow directly into the ascending aorta. In addition, the valve between the ventricle and the conus arteriosus and between the left atrium and ventricle underwent rapid hyperplasia so as to redirect blood flow away from the injured ventricle. During the time when collateral flow and valve hyperplasia prevented additional hemorrhage into the thoracic cavity, the injured ventricle regenerated.
